# Rituximab treatment for difficult-to-treat nephrotic syndrome in children:a multicenter, retrospective study

**DOI:** 10.3906/sag-2012-297

**Published:** 2021-08-30

**Authors:** Mehmet TAŞDEMİR, Nur CANPOLAT, Nurdan YILDIZ, Gül ÖZÇELİK, Meryem BENZER, Seha Kamil SAYGILI, Emine Neşe ÖZKAYIN, Özde Nisa TÜRKKAN, Ayşe BALAT, Cengiz CANDAN, Mehtap ÇELAKIL, Sevgi YAVUZ, Nurver AKINCI, Nilüfer GÖKNAR, Cihangir AKGÜN, Sebahat TÜLPAR, Harika ALPAY, Fatma Lale SEVER, İlmay BİLGE

**Affiliations:** 1 Division of Pediatric Nephrology, Department of Pediatrics, Koç University School of Medicine, İstanbul Turkey; 2 Division of Pediatric Nephrology, Department of Pediatrics, İstanbul University-Cerrahpaşa, Cerrahpaşa School of Medicine, İstanbul Turkey; 3 Division of Pediatric Nephrology, Department of Pediatrics, Marmara University School of Medicine, İstanbul Turkey; 4 Division of Pediatric Nephrology, Department of Pediatrics, Şişli Hamidiye Etfal Training and Research Hospital, İstanbul Turkey; 5 Division of Pediatric Nephrology, Department of Pediatrics, Bakırköy Dr. Sadi Konuk Training and Research Hospital, İstanbul Turkey; 6 Division of Pediatric Nephrology, Department of Pediatrics, Trakya University School of Medicine, Edirne Turkey; 7 Division of Pediatric Nephrology, Department of Pediatrics, İstanbul University İstanbul School of Medicine, İstanbul Turkey; 8 Division of Pediatric Nephrology, Department of Pediatrics, İstanbul Aydın University School of Medicine, İstanbul Turkey; 9 Division of Pediatric Nephrology, Department of Pediatrics, İstanbul Medeniyet University School of Medicine, İstanbul Turkey; 10 Division of Pediatric Nephrology, Department of Pediatrics, Kocaeli University School of Medicine, Kocaeli Turkey; 11 Division of Pediatric Nephrology, Department of Pediatrics, Kanuni Sultan Süleyman Training and Research Hospital, İstanbul Turkey; 12 Division of Pediatric Nephrology, Department of Pediatrics, Bağcılar Training and Research Hospital, İstanbul Turkey; 13 Division of Pediatric Nephrology, Department of Pediatrics, İstanbul Medipol University School of Medicine, İstanbul Turkey

**Keywords:** Frequently relapsing nephrotic syndrome, immunosuppressive agents, steroid-dependent nephrotic syndrome, steroid-resistant nephrotic syndrome, remission

## Abstract

**Background/aim:**

This study aimed to evaluate the efficacy of rituximab in children with difficult-to-treat nephrotic syndrome, considering the type of disease (steroid-sensitive or –resistant) and the dosing regimen.

**Materials and methods:**

This multicenter retrospective study enrolled children with difficult-to-treat nephrotic syndrome on rituximab treatment from 13 centers. The patients were classified based on low (single dose of 375 mg/m^2^) or high (2-4 doses of 375 mg/m^2^) initial dose of rituximab and the steroid response. Clinical outcomes were compared.

**Results:**

Data from 42 children [20 steroid-sensitive (frequent relapsing / steroid-dependent) and 22 steroid-resistant nephrotic syndrome, aged 1.9–17.3 years] were analyzed. Eleven patients with steroid-sensitive nephrotic syndrome (55%) had a relapse following initial rituximab therapy, with the mean time to first relapse of 8.4 ± 5.2 months. Complete remission was achieved in 41% and 36% of steroid-resistant patients, with the median remission time of 3.65 months. At Year 2, eight patients in steroid-sensitive group (40%) and four in steroid-resistant group (18%) were drug-free. Total cumulative doses of rituximab were higher in steroid-resistant group (p = 001). Relapse rates and time to first relapse in steroid-sensitive group or remission rates in steroid-resistant group did not differ between the low and high initial dose groups.

**Conclusion:**

The current study reveals that rituximab therapy may provide a lower relapse rate and prolonged relapse-free survival in the steroid-sensitive group, increased remission rates in the steroid-resistant group, and a significant number of drug-free patients in both groups. The optimal regimen for initial treatment and maintenance needs to be determined.

## 1. Introduction

Idiopathic nephrotic syndrome (NS) can be classified according to steroid response into steroid-sensitive nephrotic syndrome (SSNS) or steroid-resistant nephrotic syndrome (SRNS) by the International Study of Kidney Disease in Children (ISKDC) definitions [1]. The most frequent histological lesions in pediatric patients are minimal change disease (MCD) and focal segmental glomerulosclerosis (FSGS). Children with SSNS who may develop steroid dependency and/or frequent relapses by time and children with SRNS together have been recently called difficult-to-treat NS group. 

There is no consensus about treatment protocols for difficult-to-treat NS despite the presence of various recommendations [2,3]. Steroids are the primary treatment option for children with NS, which can induce remission in the majority of them. Long-term use of corticosteroids in patients with SSNS or SRNS may lead to several complications, including cataract, obesity, hypertension, and decreased bone density. The addition of calcineurin inhibitors (CNIs) or mycophenolate mofetil (MMF) could not provide long-term remission in a group of patients with difficult-to-treat NS. New steroid-sparing agents have been investigated in recent years. Rituximab (RTX), a mouse-human chimeric monoclonal antibody that binds to the CD20 antigen expressed on B cells, has become a substantial option in difficult-to-treat NS since 2004.

Rituximab has been found to be effective in SSNS patients, particularly in those who had relapses despite maintenance treatment with CNIs or MMF [4–8]. There are inconsistent results in the SRNS group on the efficacy of RTX [3,9–11]. 

Several issues remain uncertain regarding the use of RTX in the treatment of childhood NS, such as initial treatment doses, the number of infusions, the necessity of additional doses, and dose intervals [5,12,13]. Also, there are scarce data in the literature on the adverse effects of RTX in children. Besides mild infusion reactions, life-threatening conditions such as lung injury, myocarditis, and encephalitis have been reported [14,15]. 

This study aimed to evaluate RTX’s efficacy and safety at varying doses and intervals in children with difficult-to-treat NS, particularly to assess the prevention of relapses and relapse-free survival in the SSNS group and any improvement or remission in the SRNS group. 

## 2. Materials and methods

**Figure 1:**
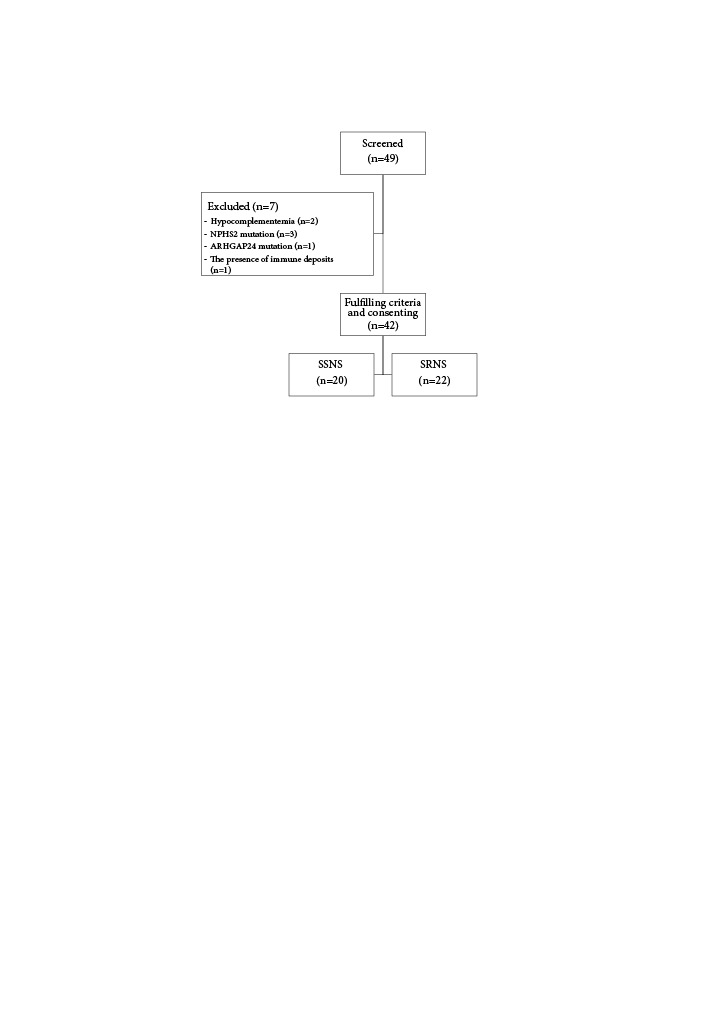
Study flow chart. Of 49 screened patients, 42 fulfilled the selection criteria and consent to study participation. Exclusion criteria were the presence of genetic mutations, hypocomplementemia, and/or immune deposits in kidney biopsy specimens. T﻿he NPHS2 gene encodes podocin that is vital for the normal glomerular filtration barrier in kidneys. NPHS2 mutation causes steroid-resistant nephrotic syndrome. ARHGAP24mutation influences podocyte cell shape and membrane structure, can causing focal segmental glomerulosclerosis. SSNS: steroid-sensitive nephrotic syndrome (including SDNS steroid-dependent nephrotic syndrome and FRNS frequently relapsing nephrotic syndrome). SRNS: steroid-resistant nephrotic syndrome.

This multicenter retrospective study enrolled children with difficult-to-treat NS on RTX treatment from 11 centers in İstanbul and two centers from the surroundings of İstanbul, Turkey. Inclusion criteria were the following: age between 1–18 years and being followed-up at least one year after RTX administration. Exclusion criteria were the presence of genetic abnormalities or secondary NS (hypocomplementemia or immune deposits in kidney biopsy specimens). The study flow-chart is shown in Figure 1. 

The patients were classified based on the steroid response; steroid-dependent nephrotic syndrome (SDNS) was defined as two consecutive relapses during steroid tapering or within 14 days of therapy cessation. Frequently relapsing nephrotic syndrome (FRNS) was defined as at least four relapses per year or two relapses within six months of the initial presentation. Steroid-resistant NS was described as no response to a 4-week course of daily corticosteroids, followed by three methylprednisolone pulses [16]. Complete remission was defined as a urinary protein/creatinine ratio <0.2 mg/mg or negative or trace dipstick on three or more consecutive days, and partial remission was defined as a urinary protein/creatinine ratio of 0.2–2 mg/mg and, if available, serum albumin level of >30 g/L [1,17]. Each center decided its own dosing regimen, including initial and maintenance doses and dose intervals of RTX. Three of 20 SSNS and 7 of 22 SRNS patients were not in the remission state at first RTX administration day. Of 11 centers, four preferred to administer maintenance doses of RTX based on B-cell depletion, seven preferred to waiting for a new relapse or a deterioration in clinical condition. Patients were subdivided into two groups according to the initial dose of RTX. The low initial dose group described the patients who received a single dose of RTX (375 mg/m^2^), whereas the patients in the high initial dose group received 2-4 doses of RTX (each dose 375 mg/m^2^) weekly or biweekly. CD19 and CD20 levels could not be evaluated because it was not available in about half of the patients in this multicenter retrospective study. 

Clinical findings, medications (including immunosuppressive use before and after RTX administration, premedication, and antibiotic prophylaxis) and laboratory parameters (complete blood count, urea, creatinine, electrolytes, albumin, urinalysis, and spot urinary protein/creatinine ratio or 24-h urinary protein excretion) were retrieved from the patient files and analyzed to evaluate treatment outcome and adverse events of RTX. Treatment success was assessed as follows: decreased or no relapses in the SSNS group, and decrease in proteinuria, increased serum albumin levels, and partial or complete remission in the SRNS group. Drug-free remission was defined as no relapse without steroids and other IS drugs, or RTX for at least 12 months. 

Standard deviation scores (SDS) of height and body mass index (BMI) were evaluated at the initial visit and annually according to Turkish children’s growth data [18]. The estimated glomerular filtration rate (GFR) was calculated with the modified Schwartz formula[19].

### 2.1. Statistical analysis

Data analysis was carried out using SPSS software version 26 (IBM SPSS Statistics, Armonk, NY, USA). The normality of the data was examined by the Shapiro–Wilk test. Continuous data were expressed as the mean ± SD if the distribution was normal and median (minimum-maximum) otherwise. The Student’s t-test was used to compare normally distributed variables, and the Mann–Whitney U test was used if data were not normally distributed. Categorical variables were presented as a number and percentage and compared using the Fisher’s exact test (independent parameters) and the McNemar test (dependent parameters). The Wilcoxon Signed Ranks test was used to compare clinical and laboratory parameters at baseline and two years in the study group. Kaplan–Meier survival analysis was used to assess the relapse-free period. Significance was allowed at p <0.05.

## 3. Results

This study included 42 children (20 SSNS (SDNS/FRNS) and 22 SRNS) aged between 1.9 and 17.3 years. There was no gender difference between the two groups, but the patients with SSNS were older at the time of the first RTX dose than the patients with SRNS (p = 0.011). A total of 37 patients had kidney biopsy; 18 showed FSGS, and 19 had MCD. The characteristics of the patients are shown in Table 1. Before using RTX, 36, 5,14, and 11 patients received cyclosporine A, tacrolimus, cyclophosphamide (CYP), and MMF, respectively. Additionally, a small number of patients received other IS agents, including levamisole or chlorambucil. Pulse methylprednisolone was administered to 85% of SSNS and 91% of SRNS patients (Table 1). 

**Table 1:** Characteristics of the patients and treatment strategies.

	SSNSN = 20	SRNSN = 22	p value
Male/Female	15/5	12/10	0.20
Age at onset of NS (years)	9.66 ± 5.16	6.70 ± 3.33	0.68
Age at the first dose of RTX (years)	12.2 ± 3.87	8.68 ± 4.09	0.011
Biopsy results, n (%)			0.004
Minimal change disease	12 (60)	7 (32)	
Focal segmental glomerulosclerosis	3 (15)	15 (68)	
No biopsy performed	5 (25)	-	
Previous immunosuppressive treatment, n (%)			
Oral steroids	20 (100)	22 (100)	NA
CNIs	19 (95)	22 (100)	0.47
MMF	2 (10)	9 (41)	0.023
Cyclophosphamide	7 (35)	7 (32)	0.82
Levamisole	1 (5)	1 (4.5)	1.00
Chlorambucil	1 (5)	-	0.47
Number of pulse steroid, n (%)			0.21
1-3 doses	12 (60)	18 (82)	
>3 doses	5 (25)	2 (9)	
Steroid toxicity*, n (%)	11 (55)	13 (59)	1.00
CNI toxicity**, n (%)	5 (25)	4 (18)	1.00
CNI dependency, n (%)	13 (65)	7 (32)	0.045
ACEI or ARB use, n (%)	9 (45)	13 (59)	0.47
Initial doses of RTX, n (%)			0.13
375mg/m2 x 1 (Low)	13 (65)	9 (41)	
375mg/m2 x 2-4 (High)	7 (35)	13 (59)	
Total doses of RTX (overall), n (%)			0.001
1-3 doses	18 (90.0)	9 (41)	
4-6 doses	2 (10.0)	13 (59)	
Number of relapses (A year before RTX use)	3.0 (1-12)	-	
Observation period (month)			
Before RTX	96.0 (14.4-187)	51.6 (4.8-122)	0.007
After RTX	30.0 (14.4-62)	31.2 (6.0-82)	0.57

SSNS: steroid-sensitive nephrotic syndrome (including SDNS: steroid-dependent nephrotic syndrome and FRNS: frequently relapsing nephrotic syndrome). SRNS: steroid-resistant nephrotic syndrome. RTX: rituximab. MMF : Mycophenolate mofetil. ACEI : angiotensin-converting enzyme inhibitor. ARB : angiotensin receptor blocker. CNI: calcineurin inhibitor. Continuous data are presented as the mean ± SD if the distribution was normal and/or median (min-max) otherwise or n (%). *Complications induced by steroid treatments, such as cataract, osteopenia, striae, hypertension, short stature, diabetes, and central obesity. **Complications induced by calcineurin inhibitors, including hirsutism, acute renal failure, posterior reversible encephalopathy, and gingival hyperplasia.

The use of a high initial dose (2–4 doses of 375 mg/m^2^) was more frequent in the SRNS group than the SSNS group (59% vs. 35%), but the difference was not statistically significant (p = 0.13). However, the total number of RTX use was significantly higher in the SRNS group than in the SSNS group (59% vs. 10%, p = 0.001) (Table 1).

The median follow-up duration after RTX was 30.0 (ranged between 14.4 and 62) and 31.2 (ranged between 6.0 and 81.6) months in patients with SSNS and SRNS, respectively (p = 0.57). The whole study group was followed for at least one year, 29 patients (15 of SSNS and 14 of SRNS group) were followed for two years or longer. 

One year after RTX administration, the median relapse rate was significantly lower than those in the last year before RTX treatment in the SSNS group (0.0 (ranged between 0 and 2) vs. 3.0 (ranged between 1 and 12), respectively).

**Figure 2:**
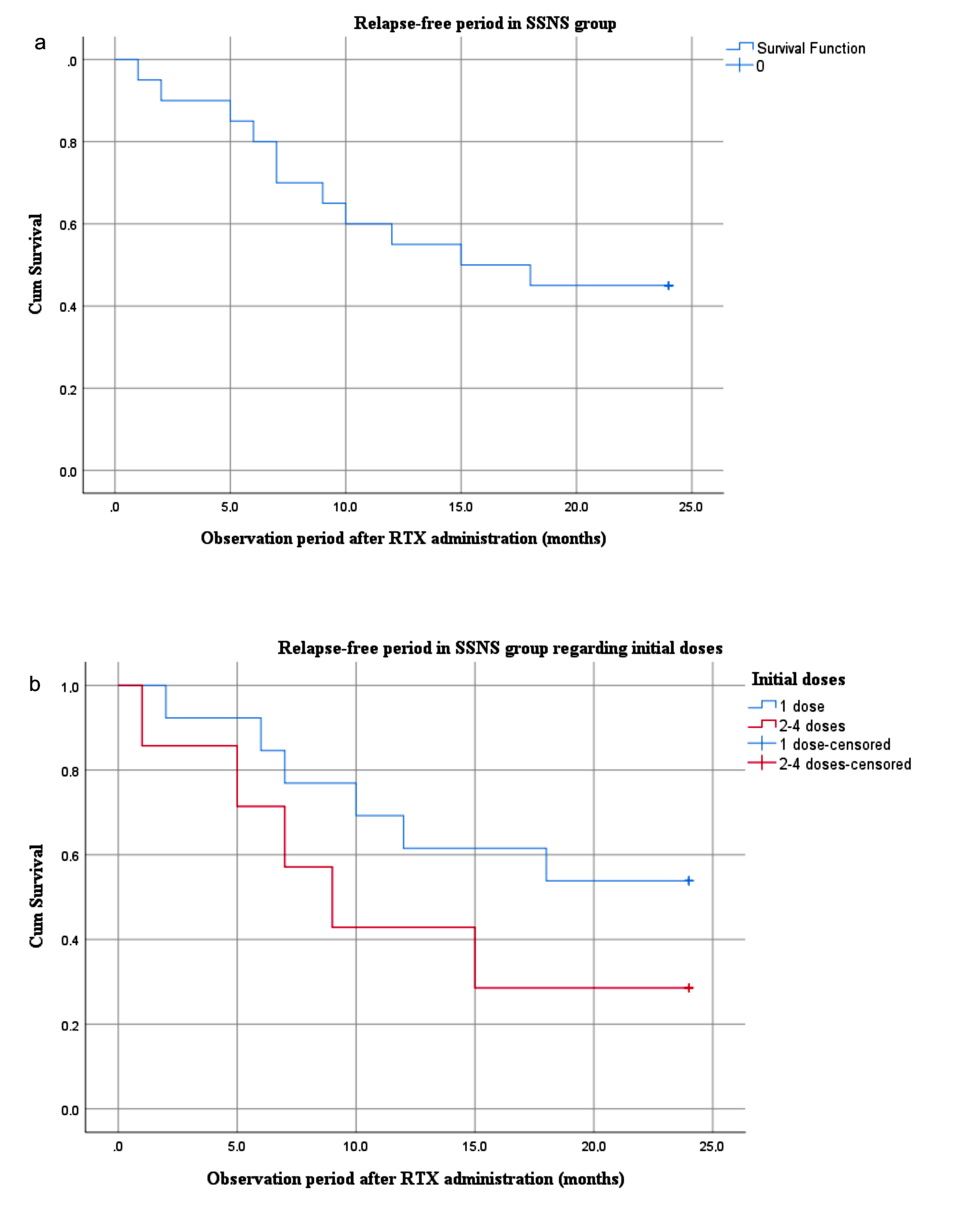
Relaps-free survival in the SSNS group (a), and adjusted for the initial dose regimens (b). SSNS: steroid-sensitive nephrotic syndrome (including SDNS: steroid-dependent nephrotic syndrome and FRNS: frequently relapsing nephrotic syndrome).

Of 20 SSNS patients, 11 (55%) had relapse following initial RTX treatment, and the mean time to first relapse was 8.4 ± 5.2 months. There was no significant difference between the low and high initial dose groups considering relapse rate (46% vs. 71%, p = 0.37) and time to first relapse (9.2 ± 5.5 vs. 7.4 ± 5.2 months, p = 0.52). In the Kaplan–Meier analysis in which 20 patients with SSNS were evaluated, the median relapse-free survival was 15 months (95% CI: 1.85–28.1), with no significant difference between low and high initial doses of RTX (p = 0.37) (Figure 2a, 2b). 

At 1- and 2-year of follow-up, serum albumin levels improved (3.25 ± 1.33 vs. 4.09 ± 0.89, p = 0.038), complete remission was achieved in 41% and 36% of SRNS patients, and partial remission in 27% and 21% of them, respectively. In the SRNS group, high initial dose therapy provided higher remission rates than the low initial dose therapy (77% vs. 56%) at the first year, but the difference was not statistically significant (p = 0.38). At the second year, the difference disappeared between the groups (57% vs. 57%). 

The median remission time was 3.65 months (ranged between 0.2 and 33.6) among SRNS patients with complete remission. Cessation or decrease of IS agents at 1-year and 2-year follow-up period in both groups was shown in Table 2. Drug-free patients were higher in the SSNS group than the SRNS group at 2 years (n = 8, 40% vs. n = 4, 18%), but the difference was not statistically significant (p = 0.26). One patient in the SRNS group did not have remission, and She/she stopped being given IS drugs due to unresponsiveness. 

**Table 2:** Immunosuppressive use at 1-year and at 2-year follow-up in the study group.

Immunosuppressives	SSNS			SRNS		
	Before RTX	1-year(N = 20) n (%)	2-year(N = 15) n (%)	Before RTX	1-year(N = 22) n (%)	2-year(N = 14) n (%)
Steroids	20	9 (45)	6 (30)	22	14 (63)	11 (50)
CNI	19	13 (65)	11 (55)	22	12 (54)	9 (40)
MMF	2	1 (5)	1 (5)	9	6 (27)	6 (27)
Drug free (at 2 years)	-	-	8 (40)	-	-	4 (18)

SSNS: steroid-sensitive nephrotic syndrome (including SDNS: steroid-dependent nephrotic syndrome and FRNS frequently relapsing nephrotic syndrome). SRNS steroid-resistant nephrotic syndrome. CNI: calcineurin inhibitor. MMF: mycophenolate mofetil. Data are presented as the n (%).

Patients received premedication with diphenhydramine and acetaminophen before RTX infusion. Trimethoprim-sulfamethoxazole prophylaxis for pneumocystis jirovecii was used only in 18 patients. Adverse events were noted mostly during infusion, such as cough (n = 5), rashes (n = 4), erythema (n = 4), mild respiratory distress (n = 3), fever (n = 2), hypotension (n = 1), nausea and vomiting (n = 1). Long-term adverse events were hypogammaglobinemia (n = 2), encephalopathy (n = 1), and myocarditis (n = 1). Mortality was observed in one case in the SRNS group with hypogammaglobulinemia due to severe pneumonia, sepsis, and dilated cardiomyopathy at 8th month after RTX. Long-term effects of RTX use were evaluated by linear growth, renal functions, and the presence of obesity and hypertension. Height-SDS, BMI-SDS, or eGFR did not change during the 2-year follow-up, both in SSNS and SRNS groups. However, the number of hypertensive patients significantly decreased in the SSNS group (p = 0.021) (Table 3).

**Table 3:** Clinical and laboratory parameters at baseline and at 2-year follow-up in the study group.

	SSNSN = 15		p value	SRNSN = 15		p value
	Baseline	2-year		Baseline	2-year	
Clinical parameters						
Height-SDS	–0.55 ± 0.99	–0.15 ± 0.96	0.31	–0.36 ± 1.03	–0.39 ± 0.88	0.75
Body mass index (kg/m2)SDS	22.7 ± 3.781.17 ± 1.12	23.5 ± 4.300.77 ± 1.21	0.460.83	20.1 ± 5.450.56 ± 0.87	19.9 ± 3.460.33 ± 1.33	0.370.51
Systolic BP (mmHg)SDS	117 ± 80.92 ± 0.79	112 ± 150.77 ± 1.22	0.400.59	109 ± 160.71 ± 1.26	110 ± 190.41 ± 1.24	0.720.46
Diastolic BP (mmHg)SDS	77 ± 101.24 ± 0.96	66 ± 80.45 ± 0.96	0.0440.11	69 ± 170.90 ± 1.32	67 ± 130.57 ± 1.08	0.680.11
Hypertension n (%)	9 (60.0)	1 (6.6)	0.021	3 (21.4)	2 (14.3)	0.25
Laboratory parameters						
eGFR (ml/min/1.7 m2)	140.4 ± 51.8	135.8 ± 44.4	0.60	143.7 ± 55.8	129.1 ± 39.8	0.80
Serum albumin (g/dL)	4.05 ± 0.68	4.25 ± 0.65	0.67	3.25 ± 1.33	4.09 ± 0.89	0.038

SSNS: steroid-sensitive nephrotic syndrome (including SDNS: steroid-dependent nephrotic syndrome and FRNS: frequently relapsing nephrotic syndrome). SRNS steroid-resistant nephrotic syndrome. SDS : standard deviation score. Data are presented as the mean±SD or n (%). The Wilcoxon Signed Ranks Test was used to compare continuous data, and the McNemar Test was used to assess hypertension frequency between the groups at baseline and at 2 years.

## 4. Discussion

The remarkable findings of this study were significant declines in the number of relapses and longer relapse-free survival in the SSNS group, notable improvement in the serum albumin levels, decrease in proteinuria and increase in the remission rates in the SRNS group, and reduced number of immunosuppressant uses in the whole series, with a low frequency of adverse events. Additionally, the number of hypertensive patients was remarkably decreased in the SSNS group. The optimal regimen for initial treatment and maintenance and long-term effects of RTX need to be determined. 

Currently, long-term follow-up data of RTX treatment in patients with difficult-to-treat NS are not available, but there are promising outcomes, particularly in the SSNS group. However, it is still unclear how many doses are necessary for initial therapy and maintenance. Even with a single dose of RTX, a significant but temporary decrease has been reported in the number of relapses in patients with SDNS [20]. 

In two recent studies evaluating children with FRNS/SDNS treated with one to four doses (375 mg/m^2^) of RTX, relapse-free survival at 1 and 2 years was achieved in 50%–70% and 41%–46% of the patients, respectively. The mean time to first relapse was found to be 9.6 and 14.6 months. The single dose of RTX has been reported to be associated with a higher relapse rate and a shorter first relapse time than those receiving 3–4 doses [21,22].

In a recently published multicenter large cohort evaluating 511 children with complicated SDNS or FRNS, low (375 mg/m2), medium (750 mg/m^2^), and high (1125-1500 mg/m^2^) doses of RTX were compared with or without maintenance immunosuppression. The authors concluded that children who received low-dose RTX without maintenance immunosuppression had the shortest relapse-free survival[23].

Consistent with previous studies, relapse-free survival was approximately 50% at 2 years in our patients with SSNS. After the first RTX treatment, more than half (55%) of SSNS patients relapsed, and the mean time of relapse was 8.4 ± 5.2 months. There was no significant difference in the relapse rate or the first relapse time between the low and high initial RTX dose groups, which can be explained by the fact that patients treated with high initial doses are likely to have a more severe clinical course than the initial low dose group. Further prospective studies with a larger sample size are needed to assess dose-related effects.

Unlike in SSNS patients, negative response to RTX has been reported in patients with SRNS. The first randomized controlled trial on RTX in children with both steroid- and CNIs-resistant NS was published by Magnasco et al. [9]. In this study, no significant difference in the degree of proteinuria was found after three months between children who received only CNI and steroids and those who received two doses of 375 mg/m^2^ RTX in addition to CNI and steroids. However, in later studies, the rates of complete or partial responses to 2–4 doses of RTX treatment in SRNS patients seem quite useful in a limited number of patients. Reported remission (complete and partial) rates range between 12.3%–76.9%, and FSGS has been associated with unresponsiveness to RTX therapy [5,10,24–27]. It is not clear that which subgroup of SRNS patients are better candidates for RTX due to heterogeneity of the underlying diseases.

In the SRNS patients included in our study group, complete remission was achieved in 41% and 36% of patients at 1- and 2-year. Considering the low and high initial dose of RTX, there was no significant difference in remission rates. Other remarkable outcomes were a significant decrease in proteinuria and an increase in serum albumin levels, and an important rate (18%) of drug-free SRNS patients at 2 years.

Since RTX was first used in idiopathic NS, many different protocols (including single dose, 2 doses and 4 doses) and different dosing practices (such as 100 mg/m^2^, 375 mg/m^2^, 750 mg/m^2^, 1500 mg/m^2^) have been reported[23]. This heterogeneity leads to difficulties in assessing the ability of different regimens. 

A single dose of RTX has been discussed in the literature, and a higher relapse rate has been reported compared to multiple doses of RTX. However, considering the duration of remission, it is also seen that many patients (75%) can be in a remission state for 4–6 months with a single dose of RTX[20, 21]. 

In a recent retrospective study, four children with SDNS and eight with SRNS received 375 mg/m^2^ RTX weekly for four weeks. The authors decided to administer RTX without considering a proteinuria-free period with steroid therapy. In this study, overall remission rates have been reported as 100% and 27% in the SDNS and SRNS groups, respectively [28]. In our study, of 20 patients with SSNS, three were no patients in remission state.

Currently, there is no consensus regarding the use of RTX with optimal initial and maintenance dose protocols. Although there is no strong evidence that lower doses increase the risk of earlier relapses or higher doses are more beneficial, the most preferred dose is 375 mg/m^2^ [29, 30]. In the present study, data were collected from 13 different centers retrospectively, with a dose of 375 mg/m^2 ^and different dose intervals (weekly or biweekly).

Children with difficult-to-treat NS, especially those with SRNS, frequently require other IS agents (including MMF or CNIs) to maintain remission. However, there are no clear suggestions about maintenance treatment after the RTX regimen. Although MMF has been reported to be effective in treating patients with SDNS, cyclosporine A is more effective in preventing relapses after rituximab [31,32]. 

Several studies reported that RTX could provide an opportunity to decrease/discontinue IS agents even with a single dose of RTX in the SDNS group for 4–6 months [20]. The percentage of drug-free patients with 1–4 doses of RTX has been reported as 24% of patients with SDNS. In recent studies, a remarkable decrease in using steroids (in 25% and 44% of the patients with FRNS/SDNS and SRNS respectively) and CNIs (in 52% and 35% of the patients with FRNS/SDNS and SRNS respectively) were noted [8, 20, 26]. In the present study, 40% of SSNS and 18% of SRNS patients were drug-free at 2 years. CNIs were the majority of maintenance therapy. 

Rituximab is usually well tolerated in children with NS. Some researchers reported no severe effects with using RTX in children with NS [21]. The most frequent adverse events are related to infusion reactions reported 5%–53% of frequency, such as pharyngeal paresthesia, cough, rash, hot flush, and fever [22]. All these events efficiently resolve with slowing infusion rate, antihistamines, and antipyretics. More serious adverse reactions were rarely reported, such as hypotension and anaphylactic reactions [33]. The mortality rate is not known but, in a 2016 article, it was reported as approximately 5% in 1 year [15]. The long-term safety of RTX in children with idiopathic NS is not known. Long-term serious complications include agranulocytosis (temporarily), lung injury, arthritis, inflammatory bowel disease, acute demyelinating neuropathy, and serum sickness disease [14,22,34–36]. Moreover, RTX increases the risk of infections, including sepsis, pneumonia, myocarditis, as well as prolonged hypogammaglobulinemia [24,37–39]. In the present study, the most frequent adverse events were rashes (19%), cough (11.9%), and mild respiratory distress during infusion (7.1%). Of 29 patients, two had hypogammaglobinemia (4.7%) in the 2-year follow-up period, one of them died due to severe pneumonia, sepsis, and dilated cardiomyopathy. 

There are few data regarding blood pressure (BP) and RTX relation. Increased BP has rarely been reported as an adverse event during infusion [6,24]. In terms of the benefit of RTX on BP, there are very limited data in the literature. Rugenetti et al. [7] reported a significant reduction in BP (particularly systolic) among 10 children with SDNS or FRNS. In our study, the number of hypertensive patients decreased in both groups, with a significance in patients with SSNS (p = 0.021). The decline in the use of immunosuppressive agents (especially steroids and CNIs) most likely account for this beneficial effect. 

This study’s strengths include the 2-year follow-up results of two patient groups with difficult-to-treat NS from 13 different pediatric nephrology centers and the assessment of BP in addition to remission, relapse rate, growth, and adverse events.

The main limitation of this study lies within its retrospective design. Obtaining data from 13 different centers, thus resulting in heterogeneity in RTX doses, intervals, and monitoring of B cell depletion, is another limitation of the present study. 

In conclusion, there is widespread interest in the use of RTX in children with difficult-to-treat NS. The results of our study based on retrospective data support the effectiveness of RTX for these children, with prolonging relapse-free survival, decreased numbers of relapse rate and hypertensive patients in the SSNS group, and increased remission rate in the SRNS group, and decreased need for immunosuppressants in both groups. Although RTX is usually well-tolerated during the short-term follow-up period, it requires close monitoring for likely adverse events that can be fatal. In terms of establishing a consensus on dosage and frequency of administration, the future well-designed/prospective studies are needed to standardize RTX treatment regimens.

## Informed consent

Written informed consent was obtained from all individual participants and their parents included in this study. All procedures performed in this study were in accordance with the ethical standards of the institutional and national research committee at which the study was conducted (IRB approval # 2019.242.IRB1.040) and with the 1964 Helsinki declaration and its later amendments or comparable ethical standards.
